# A case of adult granulosa cell tumor presenting as thoracic lesions

**DOI:** 10.1186/s44215-023-00038-1

**Published:** 2023-04-21

**Authors:** Kosuke Suzuki, Akihiko Kitami, Momoka Okada, Shinnosuke Takamiya, Shinichi Ohashi, Yoko Tanaka, Shugo Uematsu, Tetsuo Nemoto, Mitsutaka Kadokura

**Affiliations:** 1 Respiratory Disease Center, Showa University Northern Yokohama Hospital, Yokohama, Japan; 2 Department of Pathology and Laboratory Medicine, Showa University Northern Yokohama Hospital, Yokohama, Japan

**Keywords:** Granulosa cell tumor, Pleural lesions, Ovarian cyst adenocarcinoma

## Abstract

**Background:**

Granulosa cell tumors (GCTs) are uncommon sex cord-stromal ovarian neoplasms. We report a case of GCT presenting in the chest, 18 years after oophorectomy for ovarian cystadenocarcinoma.

**Case presentation:**

A 63-year-old female was admitted to our hospital for evaluation of a right hilar lesion. Subsequent abdominal and pelvic computed tomography and brain magnetic resonance imaging were unremarkable. Eighteen years earlier, the patient had undergone total abdominal hysterectomy with bilateral salpingo-oophorectomy at another institution for a uterine myoma, with the pathological findings indicating a uterine myoma, dermoid cyst of the right ovary, and serous cystadenocarcinoma and endometrioid adenoacanthofibroma around hemorrhagic cyst of the left ovary. No indication of a granulosa cell tumor (GCT) was present in either ovary. As such, diagnostic video-assisted thoracoscopic surgery was performed, revealing a well encapsulated tumor located next to the inferior pulmonary vein and small, disseminated lesions on the chest wall and diaphragm. Thereafter, we performed resection of the main tumor in its entirety and the disseminated lesions to the extent possible. Pathological examination of the resected specimens revealed a neoplasm characterized by sheets and islands of closely packed tumor cells exhibiting small follicles (Call-Exner bodies) surrounded by cells with pale, uniform nuclei, typically observed in the microfollicular pattern of adult GCT. Although the etiology of occurrence of intrathoracic granulosa cell tumor is unknown, we assumed that the neoplasm oriented from radiologically undetectable peritoneal seeding at the time of her previous surgery, with subsequent migration through the diaphragm. Over the next 10 years, she received chemotherapy with cyclophosphamide, adriamycin, and cisplatin; other combination chemotherapy; single-agent chemotherapy; and palliative radiotherapy. She died from malignant pleuritis and peritonitis 10 years after thoracoscopic surgery.

**Conclusions:**

We have reported a case of a 63-year-old female with a history of ovarian cystadenocarcinoma who underwent resection of a pleural neoplasm, which turned out to be a granulosa cell tumor. This possibility resulted from dissemination of a previous abdominal lesion.

## Background

Granulosa cell tumors (GCTs) are uncommon sex cord-stromal ovarian neoplasms with a tendency to recur several years after initial treatment. We herein report an interesting case of GCT presenting in the chest, 18 years after oophorectomy for ovarian cystadenocarcinoma. It is possible that our patient had disseminated GCT in the right thorax from undetected residual micro-foci her previous surgery. To the best our knowledge, such a presentation has not been previously reported.

## Case presentation

A 63-year-old asymptomatic female was admitted to our hospital for evaluation of a right hilar lesion by computed tomography (CT). Around 18 years prior to presentation, the patient had undergone total abdominal hysterectomy with bilateral salpingo-oophorectomy for a uterine leiomyoma at another institution, with subsequent pathology confirming a uterine myoma, a dermoid cyst of the right ovary, and serous cystadenocarcinoma and endometrioid adenoacanthofibroma around hemorrhagic cyst of the left ovary.

**Fig. 1:**
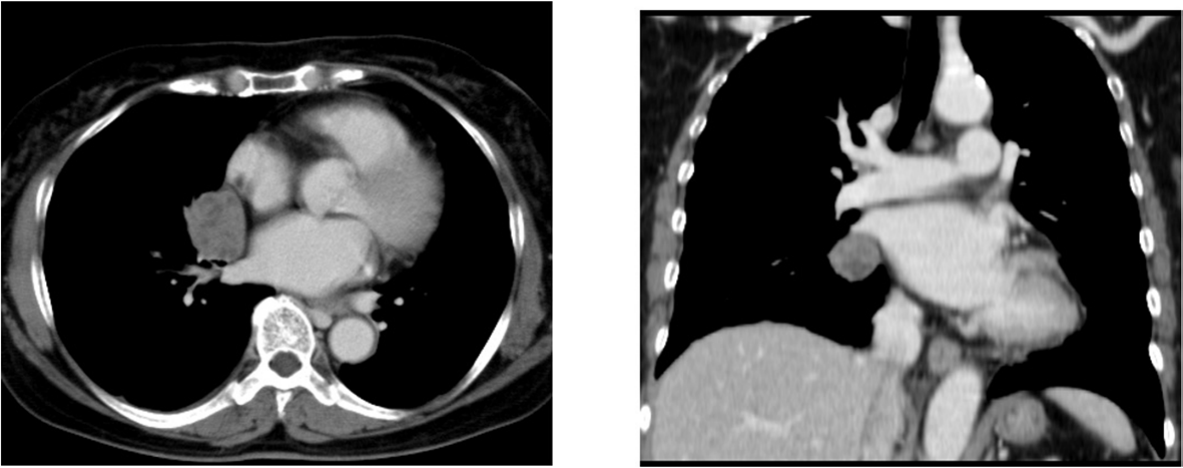
Chest computed tomography images. A well-defined lesion in the right hilum of the lung

Upon admission at our institution, the patient was obese, with a height of 151 cm and body weight of 53 kg. Her vital signs and other physical findings were unremarkable. Chest radiography demonstrated a well-defined opacity in the right hilum of the lung, whereas chest CT revealed a heterogeneous mass in the right hilum of the lung (Fig. 1), and there was no evident lesion on the diaphragm and chest wall. The results of abdominal and pelvic CT and pelvic internal examination, as well as laboratory testing on admission, were unremarkable.

**Fig. 2:**
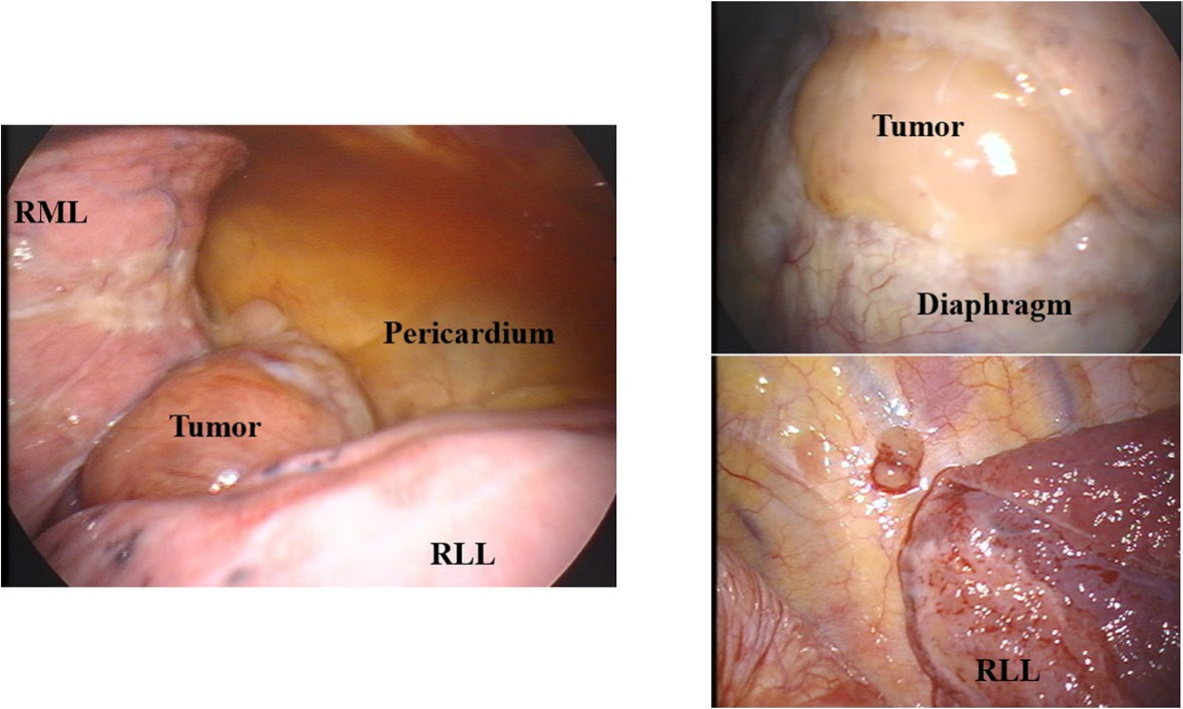
Thoracoscopic images. A tumor in the right hilum of the lung (**a**), above the diaphragm (**b**), and disseminated lesions on the chest wall (**c**)

**Fig. 3:**
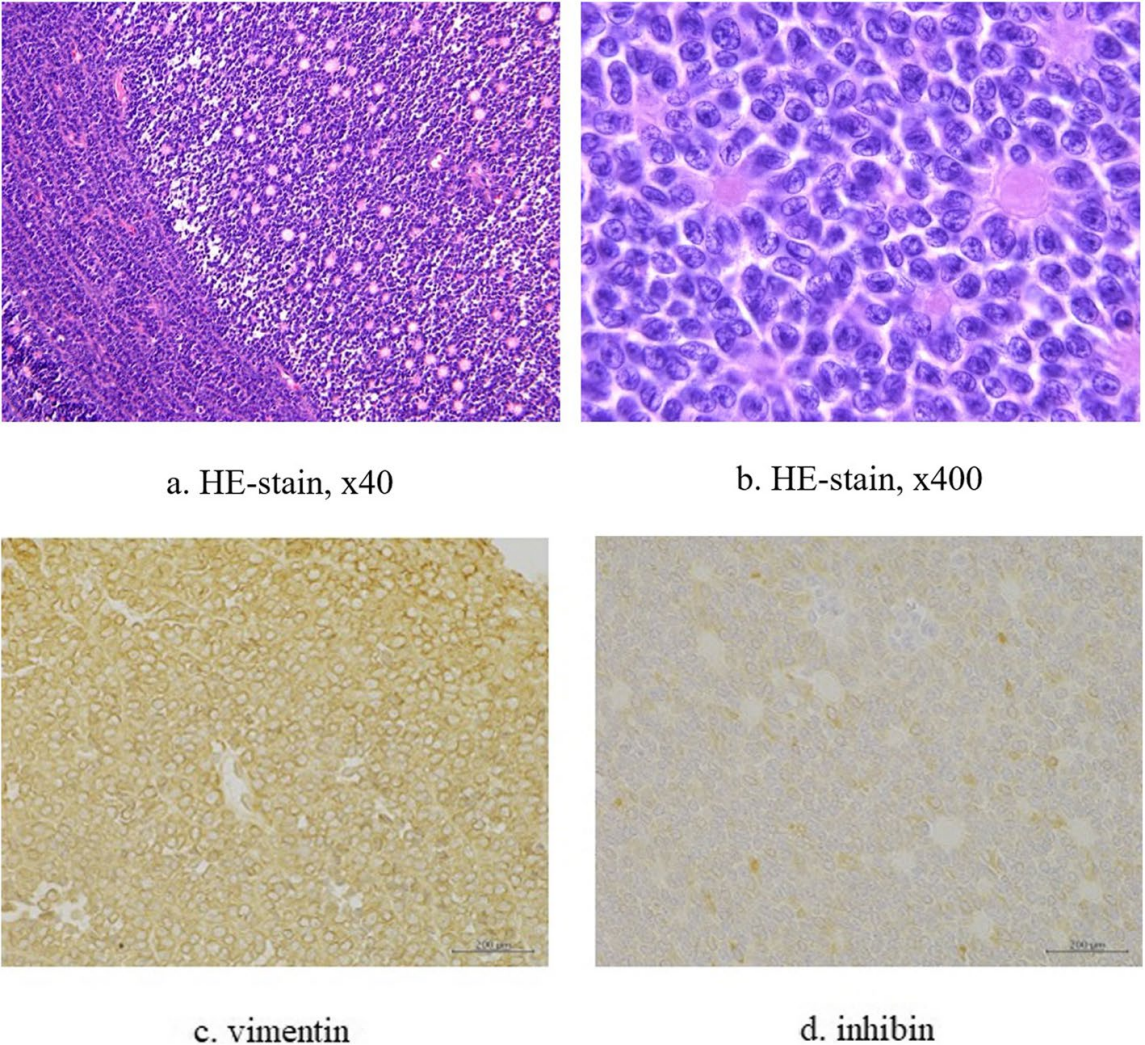
Microscopy images. Call-Exner bodies (**a**), which are small cystic areas containing cellular debris surrounded by granulosa cells with characteristic “coffee bean” nuclei (**b**). On immunohistochemistry, tumor cells stained positively for vimentin (**c**) and inhibin (**d**)

We assumed the tumor to be a mediastinal tumor, such as malignant lymphoma, thymoma, or metastatic tumor. Thoracoscopy revealed a well encapsulated mass beside the inferior pulmonary vein, nodules on the chest wall and diaphragm, and small pleural effusion (Fig. 2). At the intraoperative pathological findings, the nodules diagnosed germ cell tumor or metastatic ovarian tumor. We then performed resection of the entire main tumor and as many disseminated lesions as possible at the purpose of tumor volume reduction. The pleural effusion was determined to be cytological class II. Final pathological examination of the resected specimens revealed a neoplasm characterized by sheets and islands of closely packed tumor cells, with small follicles (Call-Exner bodies) surrounded by cells with pale, uniform nuclei (Fig. 3). On immunohistochemistry, tumor cells stained positively for vimentin and inhibin, which is typical of the adult GCT (AGCT). The pathologist at our institution reviewed the hematoxylin and eosin-stained slide from 18 years ago, though which cystadenocarcinoma of the right ovary was confirmed, with no evidence of GCT in either ovary.

Abdominal and pelvic CT, brain magnetic resonance imaging, and bone scintigraphy were unremarkable. As such, a final diagnosis of disseminated GCT, possibly resulting from undetected residual microfoci after previous surgery, was established. Thereafter, the patient underwent three cycles of cyclophosphamide (500 mg/m^2^) and adriamycin (50 mg/m^2^) with cisplatin (50 mg/m^2^) regimen (CAP).

Three years after VATS, she underwent pleural drainage and adhesion therapy with OK-432 for a massive right pleural effusion, and this procedure was repeated after 5 months for a massive left pleural effusion.

Cytological examination of the drained fluid revealed GCT cells. Collagen gel droplet-embedded culture drug sensitivity testing revealed good sensitivity of the cells to docetaxcel. The patient subsequently underwent six cycles of this treatment. A year after chemotherapy, she underwent palliative radiotherapy targeting the large mass above the right diaphragm, to which a good response was noted.

However, during her last CT at our hospital around 6 months after radiation therapy, she had developed progressive disseminated lesions in the thoracic cavity and abdomen, which promoted referral to the department of gynecologic oncology at another institution. There, she underwent seven courses of carboplatin plus paclitaxel, two courses of cisplatin plus irinotecan, and seven courses of gemcitabine plus docetaxel. Thereafter, she received single-agent chemotherapy with paclitaxel, etoposide, topotecin, and doxil. Unfortunately, her disease continued to gradually progress, and she died from malignant pleuritis and peritonitis.

## Discussion and conclusions

GCTs are uncommon sex cord-stromal ovarian tumors that account for 1–5% of all malignant ovarian neoplasms [1–3]. They have two histological types, namely AGCT and juvenile GCT (JGCT). AGCTs are common GCTs among perimenopausal and postmenopausal women, whereas JGCTs are rare and have been in premenarchal girls and young women [4]. AGCTs are characterized by low malignant potential, local spread, late recurrence, and good long-term patient survival. Although thoracic metastases from ovarian carcinoma are common, AGCTs seldom spread to the chest [5]. In fact, we found several case reports on cross-sectionally occurring lung metastases [5–12] but only three reports regarding a metastatic mediastinal lesion from AGCT [13–15].

Our patient had an AGCT presenting as thoracic cavity lesions despite having no history of this particular neoplasm. Although the etiology of occurrence of intrathoracic granulosa cell tumor is unknown, we assumed that her disseminated lesions resulted from peritoneal seeding of radiologically undetectable microfoci during her prior surgery that migrated through the diaphragm. We have several reasons for this theory.

First, thoracoscopy revealed small disseminated masses on the diaphragm and chest wall, which support seeding from the pelvis or abdomen through the diaphragm. The mechanism for this is similar to that hypothesized for catamenial pneumothorax [16]. Although cases of mediastinal AGCT lesions have rarely been reported, most recurrences are intra-abdominal. We hypothesize that radiologically undetectable intra-abdominal dissemination was present during our patient’s initial surgery, which later manifested as the lesions observed herein.

Second, the clinical course in our patient corresponds with the known pattern of recurrence observed with ovarian AGCTs. The natural history of this disease is characterized by an indolent course and a propensity for late recurrence. Cronje et al. reported that 17% of relapses occur more than 10 years after diagnosis [17].

Third, although the simultaneous occurrence of AGCT and other ovarian tumors is rare, two cases of co-existing AGCT and adenocarcinoma in the ovary have been reported [18, 19]. One instance of AGCT and dermoid cyst coexistence has also been reported [20].

Despite having reviewed the slides of ovarian specimens from the patient’s surgery 18 years prior, we could not confirm the existence of AGCT. We thought there could have been undetectable AGCT in her pelvis.

Although no standard approach has been established for the management of relapsed AGCT, surgical debulking has been the most commonly used treatment approach [5, 6]. Other treatment options, such as radiotherapy [13, 21], systemic chemotherapy [22, 23], gonadotropin-releasing hormone agonists, and aromatase inhibitors, have been used with sufficient response. Moreover, complete responses have been reported for patients receiving a combination of cisplatin, vinblastine, and bleomycin CAP [22]; and taxane plus platinum chemotherapy [23]. Our patient underwent primary surgical debulking, followed by CAP after surgery. However, 3 years after surgery, recurrence of the lesion was observed. Thus, the treatment of AGCT requires not only a multimodal approach but also careful long-term follow-up.

In conclusion, we have reported a case of a 63-year-old female with a history of ovarian cystadenocarcinoma who underwent resection of a pleural neoplasm, which turned out to be a granulosa cell tumor. The etiology of this case could be due to dissemination of previous abdominal lesions.

## Data Availability

The datasets used and/or analyzed during the current study are available from the corresponding author on reasonable request.

## Abbreviations

GCT: Granulosa cell tumors
CT: Computed tomography
VATS: Video-assisted thoracoscopic surgery
AGCT: Adult granulosa cell tumor
CAP: Cyclophosphamide and adriamycin with cisplatin regimen
JGCT: Juvenile granulosa cell tumor
